# Implementing an interprofessional information literacy course: impact on student abilities and attitudes

**DOI:** 10.5195/jmla.2018.455

**Published:** 2018-10-01

**Authors:** Marcia E. Rapchak, David A. Nolfi, Melanie T. Turk, Lori Marra, Christine K. O’Neil

**Affiliations:** School of Computing and Information, University of Pittsburgh, Pittsburgh, PA; Gumberg Library, Duquesne University, Pittsburgh, PA; School of Nursing, Duquesne University, Pittsburgh, PA; Rangos School of Health Sciences, Duquesne University, Pittsburgh, PA; School of Pharmacy, Duquesne University, Pittsburgh, PA

## Abstract

**Objectives:**

The authors investigated the impact of an interprofessional, freshman-level, information literacy course on nursing, pharmacy, and allied health professions students by examining whether students successfully met learning objectives in the course related to interprofessional attitudes, identification of research study types, and ability to relate evidence-based practice questions to their disciplines.

**Methods:**

Student posters (n=20) completed in a team project were evaluated to determine whether students were able to accurately identify the type of evidence, population, intervention, and primary outcome of studies (n=192). Additionally, posters (n=78) were evaluated to assess whether students could identify a relevant foreground question and link it to their disciplines. Students also completed the Readiness for Interprofessional Learning Scale (RIPLS) before (n=413) and after (n=352) the course to determine whether their attitudes toward interprofessional learning changed.

**Results:**

Students performed well on learning outcomes in the course, with most teams identifying relevant evidence-based practice questions (83.8%) and effectively connecting questions with their disciplines (65.4%). Students correctly identified the type of evidence, population, intervention, and primary outcome for 70.0%, 81.8%, 76.0%, and 74.0% of cited studies, respectively. Student attitudes after the course did not significantly change.

**Conclusion:**

Interprofessional information literacy education can generate positive learning experiences for freshman health care professions students to increase their beginning-level understanding of research in the health care professions and to prepare them for participation in future interprofessional courses and health care teams.

## INTRODUCTION

Interprofessional education (IPE) is important for preparing health care providers for the complexities that they will face as they work with a team to provide patient care. IPE creates “a cohesive practice between professionals from different disciplines. It is the process by which professionals reflect on and develop ways of practicing that provides an integrated and cohesive answer to the needs of the client/family/population” [1]. Instead of a fragmented approach to care, IPE shows students that a team of health care providers pools their expertise and perspectives to best serve their patients [2].

This approach to health care education was the impetus for a new, freshman-level information literacy course at Duquesne University that uses a problem-based approach to the education of future health care professionals. Using this approach, students look at a real-world scenario involving patient care or treatment rather than focus on a general search for and evaluation of health information resources. Research suggests that allied health, nursing, dentistry, and medical students are more receptive to IPE at the beginning of their programs of study, highlighting the need to introduce IPE courses at the beginning of educational programs [3].

In 2014, the university’s Information Literacy Steering Committee formed an Interdisciplinary Research and Information Skills Working Group, consisting of an instruction librarian, a health sciences librarian, and faculty members from the Schools of Nursing, Pharmacy, and Health Sciences. The members of this group developed an interprofessional, evidence-based practice (EBP) version of the required, one-credit information literacy course for incoming undergraduate freshmen, “Research and Information Skills.” While still focusing on evaluating and finding information, the IPE version of the course would ask undergraduate students from nursing, pharmacy, and health sciences (e.g., occupational therapy, speech-language pathology, physician assistant) to consider study types, understand the EBP pyramid, and find existing literature to address clinical questions. The purpose of this course would be to teach health care professions students how to search for scholarly information for their academic careers and beyond. We proposed a seven-week course that enrolled equal numbers of nursing, preprofessional pharmacy, and allied health students, all of whom begin their professional programs in the freshman year of their undergraduate careers. This course would provide an interesting and relevant introduction to information literacy for health care professions students and allow an IPE experience early in the curriculum.

In fall 2014 and fall 2015, in agreement with the Information Literacy Steering Committee, the health sciences librarian piloted 3 sections of the IPE course to demonstrate to the university the need to offer the course widely. The students who were not enrolled in these sections took the general version of the course (26 sections in fall 2014, 31 sections in fall 2015). In fall 2014, students enrolled in the IPE course rated the relevance of the course to their college studies and careers somewhat higher than health care professions or non–health care professions students enrolled in general information literacy courses [4]. Following the pilots, deans from nursing, pharmacy, and allied health, including the dean of the College of Liberal Arts, which hosted the original information literacy course, agreed to offer the IPE course as a separate course. In fall 2016 and fall 2017, 13 and 14 sections of the IPE course were offered, respectively, impacting a total of 1,133 health care professions students.

In this study, the authors evaluated whether an IPE course for freshman students in allied health, nursing, and pharmacy programs changed the students’ attitudes toward the importance of IPE using the Readiness for Interprofessional Learning Scale (RIPLS). We also examined student posters to determine whether students showed an understanding of the relevance of scholarly information for multiple health care professional disciplines and could correctly identify study types in articles that they found.

## METHODS

### Context

The IPE course is taught by seven librarians with experience in health sciences resources. This course requires students to work in an interprofessional team consisting of a nursing student, allied health student, and pharmacy student to address and find information that addresses an EBP question that follows the population, intervention, comparison, and outcome (PICO) or population, intervention, and outcome (PIO) format. Students in the course are introduced to EBP principles, including study types, that they will use in their professional careers.

Each team develops a foreground clinical question using PICO or PIO (i.e., a question designed to find evidence to inform a specific clinical decision) [5], finds background information (e.g., textbooks, reference works, websites) and primary sources, and then cites and evaluates these sources. The course learning objectives are to (1) describe how to use information ethically in academic and clinical settings, (2) access Duquesne University Library’s services and collections (electronic and print), (3) find scholarly resources needed for health care–related coursework, (4) evaluate the quality of information that they found, and (5) work cooperatively with students in other health care disciplines. Students also complete the RIPLS before and after the course as part of the course activities to determine whether their attitudes toward IPE change over the course.

For the final assignment, the student teams create posters in which they reflect on the relevance of their foreground question to each student’s discipline, which acts as a way of reemphasizing IPE attitudes, which Visser et al. encourage as a method of student-guided instruction [6]. Teams of students also describe how they searched the literature, include a table of the studies they found, and discuss the results in a poster format. They then present these posters in class and review the other teams’ posters. For example, some students address questions such as, “Are proton pump inhibitors effective in reducing symptoms of GERD in adults aged 45–70 years old?” and “Can exercise therapy increase knee strength in older adults after knee replacement?” This activity replicates the kind of communication that the students will engage in once they enter their professions and emphasizes the IPE competency of teamwork to plan and evaluate interventions, which is part of a core competency established by the Interprofessional Education Collaborative [7].

### Participants

Students enrolled in the IPE course in fall 2016 were recruited by visits from a researcher to the classes. The study was explained to students, who signed consent forms to participate in the component of the study in which their posters were examined. For the RIPLS portion of the study, students were informed of the study online and then clicked to consent to being included in the study. The 13 sections of the course had 165 allied health students, 136 nursing students, and 130 pharmacy students enrolled, representing 80% of the total population of freshman students in the health care professions programs. For allied health, nursing, and pharmacy, 82.5%, 90%, and 71% of students, respectively, were enrolled in the IPE course.

### Readiness for Interprofessional Learning Scale protocol

At the beginning and end of the semester, students took the RIPLS to determine whether the learning outcome of “Work cooperatively with students in other health care disciplines” was met. All students eighteen and older enrolled in fall 2016 were invited to participate in the assessment. The Duquesne University Institutional Review Board approved this study (protocol 2016/08/2).

The RIPLS is a validated instrument that was originally developed in 1999 to measure undergraduate students’ attitudes toward, or “readiness” to engage in, IPE activities [8]. We used an adapted version of the RIPLS that included 19 items, asking students to use a 5-point scale to indicate their agreement or disagreement with statements designed to measure their attitudes toward learning with students in other health care profession disciplines and working in teams [9]. Items 10, 11, 12, 18, and 19 were reverse-scored. RIPLS has 3 subscales: teamwork and collaboration (items 1–9, possible score range 9–45), professional identity (items 10–16, possible score range 7–35), and roles and responsibility (items 17–19, possible score range 3–15). Higher scores indicate less agreement and, thus, a more negative attitude. Cronbach’s alpha for the scale is 0.90 [8]. Example statements are “Shared learning with other health and social care students/professionals will increase my ability to understand clinical problems” and “Team-working skills are vital for all health and social care students/professionals to learn.” Related-samples Wilcoxon signed rank tests were performed using SPSS to determine whether the pre-course and post-course RIPLS subscale and total scores were significantly different.

Students in all sections of the course completed the RIPLS as part of the course, but only those who consented to being in the study were included. A total of 413 and 352 students completed pre-course and post-course RIPLS, respectively.

### Poster evaluation and assessment

To determine whether students met the learning outcome “Find scholarly resources needed for health care–related coursework,” we requested consent to examine the final posters submitted by the interprofessional student teams. All students in the 78 teams provided consent. After the end of the semester, 2 researchers examined each poster using a rubric designed to assess whether the foreground question fit the PICO/PIO framework and was relevant to the students’ health care professions: scores of 0, 1, or 2 indicated that the question was irrelevant or not in PICO/PIO format, somewhat relevant or with some PICO/PIO elements, or relevant with all PICO/PIO elements, respectively.

The students’ abilities to connect their foreground question to each of their disciplines was also evaluated: a score of 0 indicated that students did not explain the relevance of the question to any health care profession discipline, a score of 1 indicated that students explained the relevance of the question to health care professions disciplines in a basic or simple way or did not fully explain its relevance to each health care profession discipline, and a score of 2 indicated that students fully explained the relevance of the question to each health care profession discipline. For example, for the question, “Can eccentric exercise improve hand strength in adults with tennis elbow?,” physical therapy students might seek information on specific exercise regimens, nursing students might seek information on how to advise patients with tennis elbow, and pharmacy students might seek information on the use of medications in conjunction with therapy.

We randomly selected 20 of the 78 posters to assess the students’ abilities to identify key elements of the 192 studies cited. We determined that a random sample of 20 posters would be acceptable because it would represent the work of 60 students and include nearly 200 citations. Each researcher reviewed the full text of the cited articles from 4 posters and evaluated whether the students correctly identified the study type, population, intervention, and primary outcome. A correctly identified element received a score of 1, whereas an incorrectly identified element received a score of 0. For example, if the study type was a case-controlled study and the team listed it as a randomized controlled trial, they received a 0 for that element. Scores were tabulated to determine the percentage of all studies for which the teams correctly identified each element.

We also randomly selected 16 of the 78 posters to evaluate the search strategies that were used. We examined whether students used a variety of keywords in their search strategies and whether these were typical of what is found in academic literature (e.g., “adolescents” instead of “kids”).

Furthermore, student grades and withdraw rates were collected by Enrollment Management to determine whether students in the IPE course had higher grades and matriculation rates than students in the general information literacy course.

### Data analysis

RIPLS data were analyzed to determine pre-course and post-course means, standard deviations, skewness, kurtosis, and medians. Frequencies of ratings for the foreground question and the percentage of correctly identified elements of studies cited in the posters were calculated.

## RESULTS

Analysis of total and subscale RIPLS scores showed that there were no major changes in students’ attitudes toward IPE after the course (Table 1). Item subscales and total scales did not significantly differ (*p*>0.05).

**Table 1:** Pre-course and post-course subscale and total Readiness for Interprofessional Learning Scale (RIPLS) scores

	Pre-course		Post-course		Mean difference (pre- and post-)*

	Mean	Standard deviation	Mean	Standard deviation	
Teamwork and collaboration	12.79	3.71	13.03	6.76	−0.24
Professional identity	11.78	3.81	12.27	5.40	−0.49
Roles and responsibilities	6.77	1.56	6.75	2.05	0.02
Total	31.31	7.63	32.03	12.82	−0.72

* Negative difference indicates undesired shift in attitude.

Most student teams were able to identify a relevant PICO/PIO question to explore and to describe the relevance of the question to the team members’ disciplines (Table 2). Cohen’s kappa was 0.32 between the 2 raters of each poster, reflecting a fair amount of consistency [10].

**Table 2:** Ratings for foreground questions on student posters

Rating	Performance level	% of ratings	Performance level	% of ratings
0	Irrelevant: Foreground question is irrelevant to health care profession disciplines	10.3%	Not explained: Does not explain the relevance of the foreground question to any of the health care profession disciplines	9.0%
1	Relevant: Foreground question has some relevance to health care profession disciplines and some population, intervention, comparison, and outcome (PICO) or population, intervention, and outcome (PIO) elements	5.9%	Explained: Explains in a basic or simple way the relevance of the foreground question to the health care profession disciplines OR does not fully explain the relevance to each health care profession discipline in the team	25.6%
2	Very relevant: Foreground question is very relevant to health care profession disciplines and in PICO/PIO format	83.8%	Well explained: Fully explains the relevance of the foreground question to each health care profession discipline	65.4%

Students were able to correctly identify study type (e.g., cohort study, randomized controlled trial), population, intervention, and primary outcome for most of the articles cited in student posters (Table 3). However, only 3 out of 20 teams correctly identified all study elements.

**Table 3:** Percent of correctly identified study elements in student posters

Table element	% correct
Type of evidence	70.0%
Population	81.8%
Intervention	76.0%
Outcome	74.0%

Students in 14 of 16 teams (87.5%) identified alternate keywords in searching for scholarly sources, all of which were appropriate for academic research.

Approximately 84% of students in the IPE course received an A grade in fall 2016, whereas only 66% of students enrolled in the standard information literacy course received an A. Out of the 431 students in the IPE course, none received less than a C+.

## DISCUSSION

We found that health care professions students gained some understanding of EBP and IPE through their interprofessional experience. While students did not have perfect mastery over the concepts taught in the course, most (83.8%) were able to formulate a relevant PICO/PIO question and relate it to their disciplines, similar to health care professions students who completed online modules at the University at Buffalo and SUNY Buffalo State [11]. Although most teams did not correctly identify all study elements, in our judgment this is a difficult task for first-semester freshmen after they have taken a one-credit IPE information literacy course and who have been exposed to only introductory professional coursework or no professional coursework.

Although we found that a single one-credit course did not lead to significant changes in attitudes toward IPE, it did not lead to more negative interprofessional attitudes as found by Stull and Blue [12]. However, we focused on first-year preprofessional students, whereas students in Stull and Blue’s study had already completed their baccalaureate education. Students in our study began the course with positive attitudes toward teamwork, collaboration, and their professional identities, and no significant decline in these attitudes was observed after the course. However, longitudinal studies show declines in student interprofessional attitudes, especially after the first professional year [2, 13]. Since students enter their studies with positive attitudes, having an interprofessional experience in a course may make them more aware of the realities and potential challenges of interprofessional teamwork [2, 14].

There were several strengths of and limitations to this study. One strength was the large sample of students who participated. Another strength was the use of a validated tool to assess students’ readiness for interprofessional learning. A limitation was that we only collected data in one semester in which seven librarian instructors taught the course, which could have introduced inconsistency in the presentation of the materials or the emphasis on interprofessional learning.

Early introduction of IPE teamwork skills is important to create a foundation for effective teamwork in health care settings. The EBP question and poster assignment required work in teams for a common outcome, which is a key competency of IPE. Additionally, this interprofessional activity provided an introductory experience involving a team effort to solving a health-related question. Team members often provided discipline-specific contributions and information, thereby facilitating exploration of roles, another key competency of IPE. Specifically, these freshman-level health care professions students developed a stronger understanding and awareness of multiple disciplines at the very beginning of their preprofessional educational experience, providing an initial IPE experience for students and perhaps preparing them for future exposure to IPE in upper-level course work and clinical experiences.

Future research could examine whether interprofessional attitudes vary among students in different health care professions disciplines before and after the course. This could indicate whether students in particular disciplines show more positive or negative attitudes than others and could inform IPE choices for those schools. Additionally, RIPLS scores could be compared longitudinally after multiple IPE experiences to reveal whether a slight decline continues or whether attitudes become more positive as students are closer to beginning their careers.

This research can be used to improve interprofessional information literacy instruction to increase emphasis on interprofessional competencies and the importance of research skills during preprofessional education for all health care professions disciplines. IPE has been noted to enhance interprofessional collaborative knowledge, behavior, skills, and quality of patient care in a recent systematic review based on objective measurement of abilities [15]. Thus, utilizing innovative ways to initiate IPE, such as through an information literacy course at the undergraduate freshman level, is essential for positive patient outcomes. Health sciences librarians are in a key position to facilitate IPE as it relates to information literacy and EBP education [11], which ultimately promotes evidence-based patient care in the clinical arena. There is a paucity of literature on IPE initiatives that incorporate the valuable contribution of librarians. This project adds to the knowledgebase by describing the development and implementation of an IPE information literacy course for undergraduate health care professions students.
